# Mycotoxin Decontamination of Food: Cold Atmospheric Pressure Plasma versus “Classic” Decontamination

**DOI:** 10.3390/toxins9050151

**Published:** 2017-04-28

**Authors:** Nataša Hojnik, Uroš Cvelbar, Gabrijela Tavčar-Kalcher, James L. Walsh, Igor Križaj

**Affiliations:** 1 Jožef Stefan Institute, Department of Surface Engineering and Optoelectronics, Jamova cesta 39, SI-1000 Ljubljana, Slovenia; uros.cvelbar@ijs.si; 2 Jožef Stefan International Postgraduate School, Jamova cesta 39, SI-1000 Ljubljana, Slovenia; 3 University of Ljubljana, Veterinary Faculty, Institute of Food Safety, Feed and Environment, Gerbičeva 60, SI-1000 Ljubljana, Slovenia; gabrijela.tavcar-kalcher@vf.uni-lj.si; 4 University of Liverpool, Department of Electrical, Engineering and Electronics, Brownlow Hill, Liverpool L69 3GJ, UK; J.L.Walsh@liverpool.ac.uk; 5 Jožef Stefan Institute, Department of Molecular and Biomedical Sciences, Jamova cesta 39, SI-1000 Ljubljana, Slovenia

**Keywords:** cold atmospheric pressure plasma technology, mycotoxins, physical decontamination, chemical decontamination, biological decontamination

## Abstract

Mycotoxins are secondary metabolites produced by several filamentous fungi, which frequently contaminate our food, and can result in human diseases affecting vital systems such as the nervous and immune systems. They can also trigger various forms of cancer. Intensive food production is contributing to incorrect handling, transport and storage of the food, resulting in increased levels of mycotoxin contamination. Mycotoxins are structurally very diverse molecules necessitating versatile food decontamination approaches, which are grouped into physical, chemical and biological techniques. In this review, a new and promising approach involving the use of cold atmospheric pressure plasma is considered, which may overcome multiple weaknesses associated with the classical methods. In addition to its mycotoxin destruction efficiency, cold atmospheric pressure plasma is cost effective, ecologically neutral and has a negligible effect on the quality of food products following treatment in comparison to classical methods.

## 1. Introduction

Many species of filamentous fungi have the ability to produce toxic secondary metabolites known as mycotoxins. The term mycotoxin is used only for toxic substances produced by fungi related to food products and animal feed; it does not include toxins produced by mushrooms [1]. Today, about 400 structurally different mycotoxins have been discovered and divided into the following main groups: (i) aflatoxins produced by *Aspergillus* species and ochratoxins produced by both *Aspergillus* and *Penicillium* species; (ii) trichothecenes, zearalenone and fumonisins produced by *Fusarium* species; and (iii) ergot alkaloids, produced by *Claviceps* species, and others [2]. Generally, mycotoxins represent a significant threat to human health as they can be carcinogenic, neurotoxic and toxic to the endocrine or immune system [3]. They can appear in the food chain due to infected crops, which are either consumed directly by humans or used as livestock feed, appearing in meat, milk or eggs. Beside this, they can contaminate food such as cereals, fruits, nuts, spices and other by-products as seen from Table 1 [4].

Today, the trend of mycotoxins food contamination is increasing to alarming values with 25% of cereals worldwide already unsuitable for consumption [5]. Undesirable fungal growth and mycotoxin production is usually a result of incorrect agricultural and harvesting practices as well as the low effectiveness of prevention methods [3]. To reduce the potential danger to human health, many countries worldwide adopted strict legislation to control the mycotoxin presence in food and feed. In European Union, the presence of mycotoxins in food and feed is regulated by Regulation (EC) No 1881/2006, Directive 2002/32/EC, Recommendations 2006/576/EC and 2013/165/EU, and their amendments [6,7,8,9]. On top of this, recent studies have revealed a correlation between the increased presence of mycotoxins and global climate change [10]. Parameters including elevated temperatures, moisture levels and plant stress-related response stimulate fungal growth and, consequently, production of mycotoxins [10,11,12,13]. Furthermore, climate change plays a significant role in the global economy, where food is transported over long distances from producer to consumer, and may be subject to different local climates, transport and prolonged storage times. All these factors may contribute to increased food contamination [14].

Looking forward, it is expected that by the year 2050 the human population will exceed 9.2 billion. This will place an additional and unprecedented burden on the global food supply chain. The combination of modified climatic conditions and a tendency for consumers to eat healthier and fresher foods makes it imperative that new, sustainable and more effective approaches in agriculture, processing, transportation, and storage methods are developed. New mycotoxin-decontamination technologies will play a role in all stages of the supply chain. Beside this, the novel methods will have to preserve the quality of food products, be environmentally benign and economically suitable [14].

**Figure 1:**
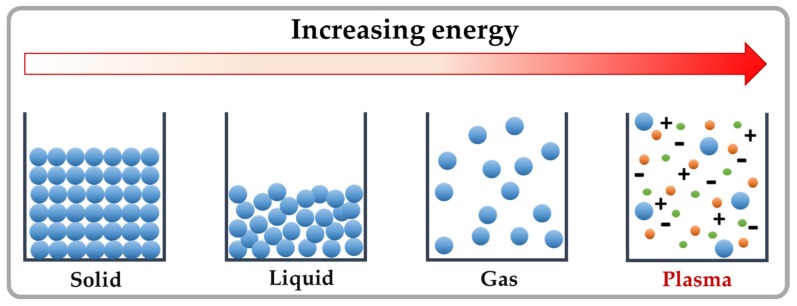
The generation of plasma: by adding energy to material, gas of electrons and ions is eventually produced. This fourth state of matter is referred to as “plasma”.

Considering the above-mentioned requirements, cold plasma technology represents a promising non-thermal mycotoxin-decontamination approach. Plasma is generally known as the fourth state of matter; a plasma state is reached by increasing the energy level of a substance from a solid state through the liquid and gaseous states of matter, ending in an ionized state of gas, which has unique physical and chemical properties (Figure 1) [15]. In electrically created plasmas, energy is delivered in the form of an electric field from an electrical power source; seed electrons produced by UV or background radiation are accelerated by the applied electric field leading to the excitation, dissociation or ionization of the background gas. Ionization, caused by the collision of an energetic electron with a neutral atom or molecule, results in the production of further electrons which are also accelerated in electric field. These free and energetic electrons subsequently collide with other surrounding molecules and atoms present in the gas, resulting in an avalanche process. Through the simultaneous generation and interaction among electrons, neutrals, metastables and ions, a vast number of reactions occur, yielding a wide variety of reactive chemical species [16,17]. In complex gas mixtures, such as humid air, a large number of reactive chemical species are created which take part in many hundreds of reactions [18]. In addition, molecules or atoms in an exited state can emit photons with wavelengths in the UVC, UVB and UVA range [19].

Plasma can be produced under low pressure or even atmospheric pressure conditions. Typically, low-pressure plasma systems require a discharge generator, a gas source and an expensive vacuum system, consisting of pumps and vacuum chamber. Such systems are widely used for applications in material processing. Nevertheless, they are not suitable for materials sensitive to low-pressure conditions including biological material [17]. The use of atmospheric pressure plasma avoids the disadvantages of vacuum systems and enables the treatment of biological materials. Common examples of atmospheric pressure plasma systems include arc, corona and dielectric barrier discharges [20]. The most perspective discharges for the treatment of biological materials are those that are in thermal non-equilibrium, having a gas temperature that is close to room temperature, and are typically referred to as cold plasmas. Recent developments in cold atmospheric pressure plasma (CAP) sources and the ability to tailor discharges to produce highly reactive species in high concentrations, but at temperatures close to room temperature, have paved the way for a wide number of biological applications. Such CAPs are also suitable for use in electronics, surface modifications in polymer and textile industry, synthesis of nanoparticles and degradation of pollutants [21]. The new findings and developments in plasma science through the last decade reveal the great potential of CAP as an innovative technology in the field of biology, moving from the treatment of inanimate materials to living or cellular objects. Such applications include CAP treatments in medicine as well as in agriculture and the food industry. In the area of plasma medicine, research is mostly focused towards CAP use for skin treatment and wound healing, cancer cell and tumour treatment, dental implant sterilization, bone growth and many others [22].

CAP technology is, on the other hand, a newcomer to the field of agriculture and food industry. The main advantage of the CAP treatment of food products refers to its high chemical reactivity, achieved through the reactive species generated, and consequently its ability to deactivate harmful agents such as pathogenic bacteria and toxic pollutants in short processing times and at low temperatures with almost negligible impact on the treated food products. The technology can be applied to different types of food products in both solid and liquid form. In addition, the low energy consumption of such discharges and price-value inputs contribute to CAP being considered as an economically acceptable method [16,21,23,24]. Considering this, CAP technology also has a high potential as a decontamination tool for both mycotoxin-producing fungi and the mycotoxins that they produce. Different set-ups in both low and atmospheric pressure conditions have been used on mycotoxins such as aflatoxin B_1_, deoxynivalenol and nivalenol (AFB_1_, DON, and NIV, respectively) resulting in high decontamination rates in only a matter of seconds [25,26,27]. Treatments of the mycotoxin-producing fungi, e.g., one of the main mycotoxin producers *Aspergillus* spp. and *Penicillium* spp., with plasma, demonstrated very promising results as well. Plasma was able to stop or significantly reduce the further growth of fungi on different contaminated food products including corn, bean, cereals, fruits, nuts and many others [28,29,30,31,32,33]. Perhaps even more importantly, plasma treatments did not significantly influence the organoleptic characteristics of the treated foods or their nutritional properties [27,33,34,35]. Despite promising results, the use of CAP in the field of mycotoxin decontamination needs further exploration to uncover the CAP-related decontamination chemical processes and to develop optimised plasma systems suitable to meet the requirements of food processing and safety.

The aim of this contribution is to critically review plasma technology as a new food processing approach in the field of mycotoxin decontamination. The main advantages of this method over the classical mycotoxin-decontamination methods are considered and compared.

## 2. The Background of CAP Decontamination in Agriculture and Food Industry

A key benefit of the use of CAP technology in the field of agriculture and food is its high decontamination efficiency, which can be achieved in short treatment times and in non-thermal conditions. Its applicability has been widely demonstrated by successful decontamination of food with bacterial pathogens (*Escherichia coli*, *Salmonella typhimurium*, *Staphylococcus aureus*, *Listeria monocytogenes*, etc.) and harmful compounds (phenolic compounds, pesticides, azo dyes, etc.) [21,35,36,37,38,39,40,41,42,43]. The mechanisms of CAP decontamination are attributed primarily to the highly reactive oxygen and nitrogen species (ROS and RNS) created within the plasma as well as UV radiation, which induce highly oxidizing effects [22,24,44,45,46]. 

**Figure 2:**
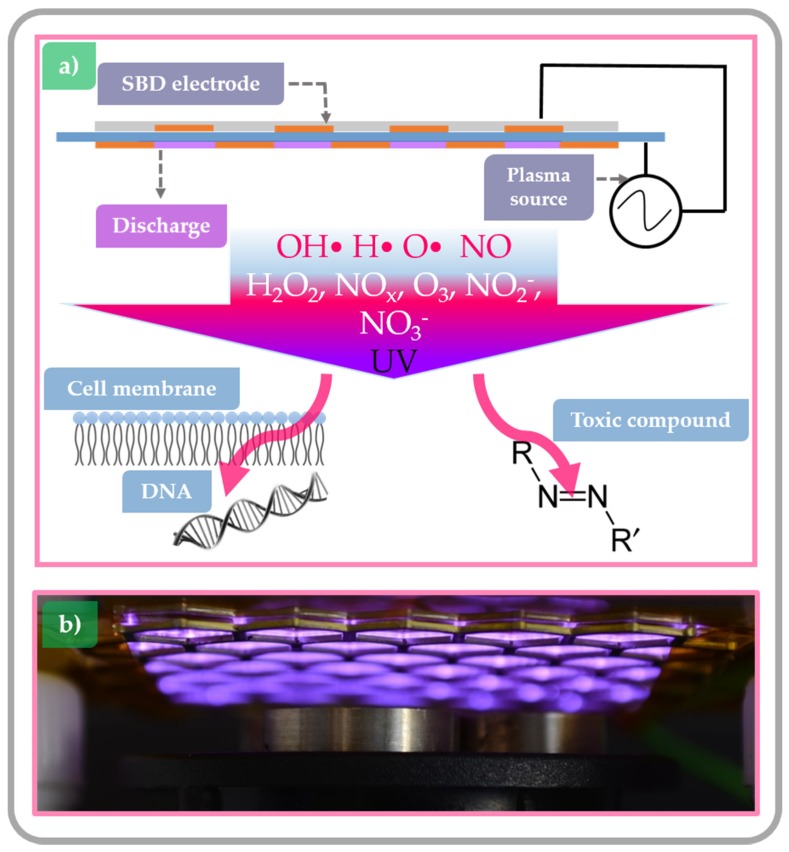
(**a**) Scheme of an air surface dielectric barrier (SDB) CAP set up; and (**b**) photo showing the CAP SDB system used in the presented experiments.

The prevalent primary species in an air plasma include radicals such as OH•, H•, O•, and NO. These radicals can react with each other, and with the ambient/background gas (air), vapour or even liquids, where they create oxygen- and nitrogen-based secondary species such as H_2_O_2_, NO_x_, O_3_, NO_2_^−^, NO_3_^−^, peroxynitrite, etc. These plasma species can be divided into short- and long-lived species depending on their lifetime [18]. Long-lived species can also exist after the plasma source is removed or turned off resulting in post-discharge reactions, which is named the plasma afterglow [44]. The importance of these species can be observed after primary interaction such as in the case of living cells, where these species first react with the cell plasma membrane, and later can enter the cell and cause damage to intercellular elements, such as organelles and biomolecules such as DNA, RNA and proteins. Similarly, when toxic compounds are exposed to the ROS and RNS produced in the plasma, they are decomposed directly or indirectly through secondary chemical processes with the transformation of toxic substances into less toxic reaction products (Figure 2) [47].

Among all plasma species, many studies have highlighted the key role played by atomic Oxygen (O), hydroxyl radical (OH•), ozone (O_3_), hydrogen peroxide (H_2_O_2_) and peroxynitrite in CAP-related decontamination effects, since they all possess a very high oxidative potential. In biological systems such as bacterial and fungal cells, the short-lived O and OH• first react with cell walls and membranes and with all the compounds composing these two structures (lipids, proteins and polysaccharides). The lipids are the most sensitive to oxidation. The mechanism of OH• reaction with lipids refers to its H-abstraction from the unsaturated carbon bonds of the fatty acids, ending in lipid peroxidation [44]. O_3_ is also a powerful oxidant; ozonation alone represents one of the most potent sanitizing and detoxifying approaches in the food industry and mycotoxin decontamination. O_3_ has high reactivity, penetrability, and spontaneous decomposition into non-toxic oxygen without forming harmful oxygen species. Compared to OH•, O_3_ induced reaction kinetics are slower [48]. In addition, the antimicrobial activity of H_2_O_2_ is well explored. Generally, cytotoxicity caused by H_2_O_2_ begins with penetration into cells and then transformation to OH• through Fenton’s reaction causing intercellular damage [49]. Peroxynitrite has recently been the object of many studies as it has been found to play an important role in oxidative stress and various diseases (neurodegenerative diseases, AIDS, arteriosclerosis, etc.) [50]. It oxidizes biomolecules directly or through H^+^- or CO_2_-catalysed homolysis. As for direct reactivity, it has affinity on key parts in proteins such as thiols, iron/sulphur centres, and zinc fingers. The lifetime of peroxynitrite is relatively short, nonetheless, it can still cross membranes and reach deep within the cell, which allows it to interact with most of the important biomolecules [51,52]. Regarding the CAP decontamination of toxic compounds, OH• as one of the strongest oxidative species initiates the toxic molecule oxidation, resulting in its degradation. However, other slower reaction pathways such as those caused by O_3_ and H_2_O_2_ are shunted or even bypassed [43].

**Figure 3:**
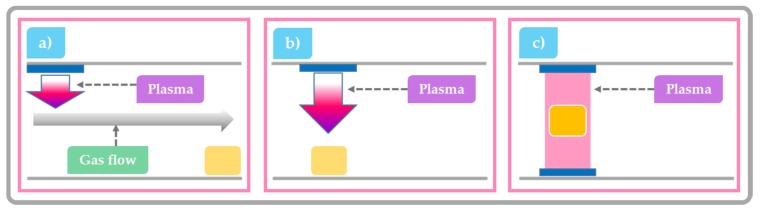
Schematic overview of common CAP systems considered for use in the food industry: (**a**) remote treatment where the sample is physically separated from the plasma generation point (**b**) semi-direct exposure, where the sample is placed close to the plasma generating electrodes; and (**c**) direct-exposure, where the sample is positioned between the plasma generating electrodes.

Plasma species production strongly depends on the CAP system design and its mode of operation. When building a plasma system for food processing, there is a wide range of operating or so-called discharge parameters to choose from, including different gasses (air, O_2_, N_2_, He, Ar, etc.) and gas flows, discharge types, discharge volumes, electrode setups, etc. The discharge can be generated using high-voltage electrical power sources or intense laser light [46]. In general, these systems can be divided into three groups defined by the position of the treated food product with respect to the point of plasma generation: at some significant distance from the generation point, relatively close to generation point or within the plasma discharge itself. With a change in position of the sample with respect to the plasma, the nature and flux of chemical species varies significantly and result in different surface effects [53]. The first category refers to remote treatment with CAP where the sample is physically separated from the plasma generation point. In this scenario, the plasma generated species are usually transported to the sample by diffusion or by an induced flow. By the time plasma species reach the targeted surface, they are mostly composed of longer-lived plasma species, with a negligible concentration of highly reactive species (Figure 3a) [54]. The second category of system enables a semi-direct treatment with CAP. Then the target is exposed to higher concentrations of short-lived and highly reactive chemical species due to the relatively short distance between plasma generation point and substrate. In this scenario, the flux of UV photons reaching the targeted surface is also relatively high (Figure 3b) [55]. The last category is known as a direct contact system where the sample is placed between the electrodes of the plasma generation system and is consequently bombarded by large fluxes of reactive species and UV light (Figure 3c) [56,57]. While direct treatment should offer the highest possible degradation and decontamination efficacy, its implementation is problematic. The sample forms part of the electrical circuit and its presence can disrupt the discharge leading to the formation of hot spots that can damage the product.

A wide range of design elements and discharge parameters enable a high degree of flexibility when designing CAP systems for food processing purposes no matter the type, size, and shape of the treated food products [58]. In terms of mycotoxin removal, plasma technology has mostly been used for the treatment of seeds, cereals, crops and fresh products [30,34,58,59]. For example, the use of an atmospheric pressure fluidized bed plasma system with air and nitrogen as a feed gas, which was used for the inactivation of *A. flavus* and *A. parasiticus* contaminated maize resulting in a 5.48 log reduction [28]. Furthermore, the production of fumonisin B_2_ and ochratoxin A (FB_2_ and OTA, respectively) was inhibited after the exposure of *A. niger* on date palm fruits to an argon CAP source. Oxygen CAP was applied for the treatment of *C. cladosporioides* and *P. citrinum* on the surface of dried filefish fillets reducing the fungi by more than 90% [33]. Moreover, argon and oxygen CAP proved to be efficient against *A. brasiliensis* contaminating pistachios [60]. Siciliano et al. performed CAP treatment with different mixtures of oxygen and nitrogen for decontamination of AFB_1_ from dehulled hazelnuts succeeding 70% decontamination rate [27]. One of the most interesting CAP applications is also the in-package treatment of food. In this scenario, strawberries were treated with CAP generated between the electrode gap and inside a sealed package. The background microflora containing fungal species was reduced by 2 log reduction which could significantly prolong the food product expiry date [35].

## 3. The Comparison of “Classic” Approaches and CAP Technology in the Field of Mycotoxin Decontamination

**Figure 4:**
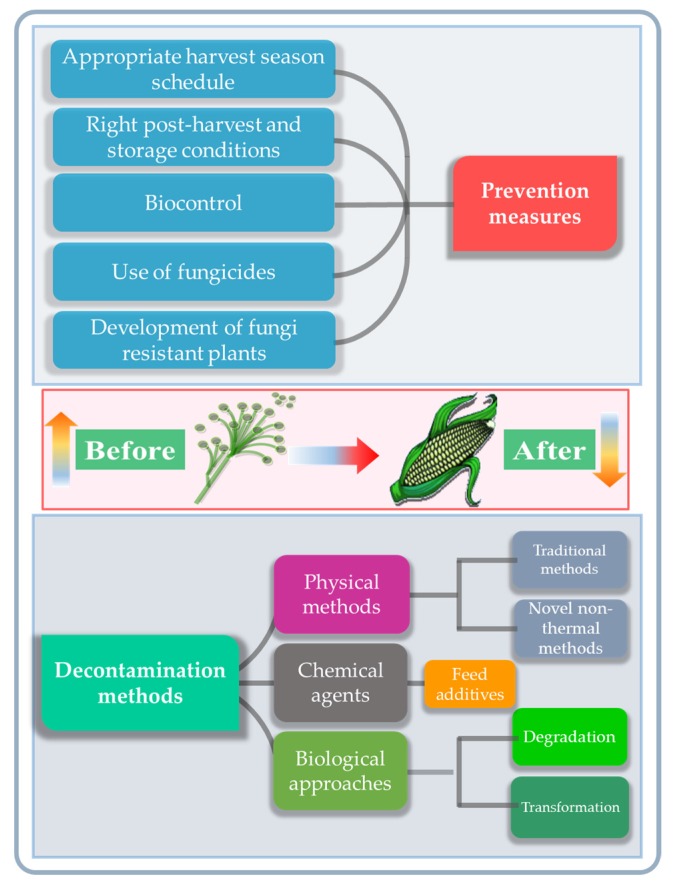
Overview of the currently available mycotoxin prevention and decontamination measures taken before and after fungal and mycotoxin contamination of food.

Actions for preventing the fungal and mycotoxin contamination of feedstuff are performed at critical points before the expected fungal infestation. This may occur at the pre-harvest stage, during the harvest-time or at the post-harvest handling and storage stages [61]. The most effective approach is primary prevention, which should be carried out before the fungal invasion and mycotoxin production occurs. Current approaches include the use of fungicides to inhibit fungal growth, an appropriate scheduling of harvesting, and maintaining the optimum storage conditions after harvest [62,63]. Unfortunately, such techniques are not entirely effective and the efficiency of fungicides varies for different fungal species [64]. For this reason, several recent approaches have focused on the development of fungi-resistant plants [65]. The various physical, chemical and biological methods for the reduction of mycotoxin contamination currently in use or under active investigation for food and feed products are reviewed below, and compared with CAP technology (Figure 4) [61].

Methods for the physical decontamination of contaminated food are typically divided into traditional measures and novel non-thermal methods. The first group refers to methods that include sorting, washing, dehulling, density segregation, grain milling and thermal treatment. The principle of their decontamination is mostly based on the removal of the contaminated food parts and consequently the mycotoxins [66]. On the contrary, thermal treatment causes thermal degradation of mycotoxins [67,68,69]. Most of the traditional methods can reach satisfying decontamination rates for different types of toxins [2,70]. However, the processing time is usually very long, requiring high energy input, and is therefore very expensive. In addition, heat treatment can significantly affect the quality of the treated food products [61]. For these reasons, the food industry is looking to further develop new non-thermal approaches such as UV- and gamma-irradiation, pulsed-light treatment as well as CAP technology. Non-thermal methods typically affect the chemical structure of the mycotoxins leading to their degradation. Their decontamination efficiency depends on the presence of water in the treated food products, the extent of mycotoxin contamination, and the intensity of exposure [66]. For gamma irradiation technology, it has been reported that the decontamination of various mycotoxins was significantly more successful when they were present in solution, reaching up to 90% removal rate. The dosages of gamma irradiation used were from 1 to 20 kGy. Degradation in this case was probably a result of the formation of free radicals which were produced by the radiolysis of water. On the contrary, gamma irradiation decontamination of the mycotoxin-contaminated solids and dry food products in conditions with low moisture values was notably less effective [71]. High doses of gamma irradiation could, however, negatively affect the quality of food products such as grains and seeds, reducing their germination ability for example [72]. UV light irradiation also demonstrated high efficiency in mycotoxin decontamination. Treatments using a wavelength of 365 nm were capable of reducing the content of aflatoxins (AFs) from various types of nuts by more than 90% [73]. The 365 nm light irradiation was capable of removing the AFB_1_ in peanut oil almost completely. Moreover, toxicity tests employing human embryo hepatocytes showed a significant reduction of toxicity of the degradation products [74]. When an AFB_1_ aqueous solution underwent the UV light irradiation treatment, three major degradation products were observed (Table 2). It was indicated that the UV light irradiation probably interacted with most of the active sites on AFB_1_, i.e., C_8_-C_9_ and O_1_-C_14_ bonds. These two bonds have been recognized as being responsible for the AFB_1_ toxicity and were transformed to more stable saturated bonds [75]. Interestingly, the structure of AFB_1_ degradation products depended significantly on the media. Different degradation products were identified when the treatment was performed in acetonitrile or in peanut oil solution (Table 2) [74,76]. UV light irradiation was also used for patulin (PAT)-contaminated apple juice. Performed at 222 nm, a 90% reduction of mycotoxin content was achieved. However, such treatments lowered the concentration of some other photosensitive substances in the juice, including healthy ascorbic acid [77]. Similar to UV light irradiation, Moreau et al. studied mycotoxin, OTA, ZEN, DON or AFB_1_, decontamination with pulsed light. The light flashes used were 300 μs in duration with a broad spectrum of light ranging from 180 to 1100 nm and a light flux 1 J/cm^2^. The analysis demonstrated that eight light flashes almost completely removed mycotoxins from the solution. Remaining toxicity was assessed on nematode *Caenhorhabditis elegans*. Degradation products of DON and ZEN were evaluated as not toxic. The mutagenic activity based on an Ames test showed that AFB_1_ degradation products were not mutagenic [78]. In a recent study, a pulsed light system of 0.52 J/cm^2^/pulse and 360 μs long flashes, with wavelengths ranging from 100 to 1100 nm were used to decontaminate AFB_1_ and aflatoxin B_2_ (AFB_2_) on different rice products. Decontamination efficiency higher than 90% was achieved for AFB_1_ after 15 s of treatment in the case of rice bran [79].

An alternate detoxification strategy employs chemical agents, which are able to detoxify mycotoxins when added to a contaminated feedstuff. The effect is achieved by many synthetic and naturally occurring compounds including various organic acids, ammonium hydroxide, calcium hydroxide mono-methylamine, hydrochloric acid, hydrogen peroxide, bisulphite, chlorinating agents, formaldehyde, ammonia, clove oil and many more [63]. Ammoniation is conventionally used for AFs decontamination of feed such as cottonseed and peanut meal. The effectiveness of detoxification with ammonia increases with the quantity of ammonia used, the time of treatment, the temperature and pressure level [80]. Many types of ammoniation are available, with the two most commonly used being high-pressure/high-temperature treatment and atmospheric pressure/moderate temperature treatment. Both methods are able to reduce mycotoxin content up to 90%. For example, the degradation of AFB_1_ by ammoniation is accomplished by hydrolysis of the lactone ring, which is followed by decarboxylation to AFD_1_ and subsequent loss of cyclopentane ring (Table 2) [81,82,83]. Ozonation is a rather new way of mycotoxin decontamination in food processing [48]. When AF-contaminated corn flour was exposed to 75 mg/L of ozone for 60 min, the content of AFB_1_, aflatoxin G_1_ (AFG_1_) and AFB_2_ decreased from 53.60, 12.08 and 2.42 μg/kg to 11.38, 3.37 and 0.71 μg/kg, respectively [84]. In another study, 89.4% decomposition of AFB_1_ was achieved after AFB_1_-contaminated peanuts exposure to ozone of 50 mg/L at a flow rate of 5 L/min for 60 min. Following this results, the two most probable AFB_1_-degradation pathways were proposed. In the first, the ozone initially reacts with a C_8_-C_9_ double bond of the furan ring in AFB_1_ in electrophilic reaction based on Criegee mechanism, whereas the second degradation pathway starts with oxidation of the AFB_1_ benzene methoxy group. Both reaction pathways lead to five final degradation products (Table 2). Generally, the toxicity of most degradation products is reduced under the assumption that a C_8_-C_9_ double bond represents one of the sites responsible for toxicity of AFB_1_ [85]. Wang et al. used ozone to achieve a reduction in toxicity of DON contaminating wheat grains. After 60 min of 100 mg/L ozone treatment, the concentration of DON decreased from 3.89 mg/kg to 0.83 mg/kg, which is under the generally recognized maximum mycotoxin limit in feed [86]. Another method of chemical mycotoxin decontamination is the use of feed additives. These inorganic and organic mycotoxin binders are added to a feedstock when there is an indication of mycotoxin contamination. Typical additives include clays as natural mycotoxin adsorbents made of silicates or aluminosilicates. The level of adsorption of mycotoxins depends on the size and the charge of the mycotoxin with regard to the specific structure of the clay used [61]. The majority of clays are able to bind AFs, but not ZEN, fumonisins and trichothecenes, when added at a concentration of 10 g/kg [87,88]. On the other hand, bentonite was able to adsorb T-2 toxin, but to achieve high binding efficiency much more than 10 g of adsorbent per kg had to be used [89]. Inconveniently, clays are also able to adsorb the micronutrients from feed and disturb the bioavailability of minerals and trace elements. Furthermore, the contamination of clays with dioxins is possible [90]. As the inorganic adsorbents proved to be inefficient removers of the majority of mycotoxins, natural eco-friendly organic binders have been introduced instead, including oath fibres and cell extracts of lactic acid bacteria and *Saccharomyces cerevisiae* [91,92]. The decontamination of food by chemical means may be inexpensive and can achieve good decontamination results; however, most of these methods can present a risk for the environment as well as for human health. A further disadvantage is the long treatment time, which is not good for preservation of high quality foods.

The final category of mycotoxin decontamination measures includes biological methods. These procedures are based on the ability of microorganisms such as bacteria, yeast, moulds, actinomycetes and algae to remove or degrade mycotoxins in food and feed products. A clear advantage of biological decontamination approaches is that no chemicals are involved. The methods are based on biological transformation, enzymatic degradation, or modification of mycotoxins to less toxic substances. Mycotoxins can be thus acetylated, glucosylated, cleaved at their rings, hydrolysed, deaminated or decarboxylated [93]. Microorganisms capable of mycotoxin detoxification include species such as *Bacillus* spp., *Brevibacterium* spp., *Pseudomonas* spp., *Rhodococcus erythropolis*, *Aspergillus* spp., *Rhizopus* spp. and *Trichosporon mycotoxinivorans*. They can efficiently detoxify a wide range of mycotoxins including AFB_1_, AFG_1_, OTA, ZEN, PAT and DON [94,95,96,97,98,99,100,101,102,103]. In addition to reducing bioavailability of mycotoxins, some microorganisms, e.g., probiotic bacteria, are frequently added to feed as an additive with positive effect on the gut flora. The effectiveness of such microorganisms to act anti-mycotoxically largely depends on their ability to remain stable in the gastrointestinal tract. The main representatives of this group of bacteria are lactic acid bacteria [104]. Generally, biological approaches are not expensive and their environmental impact is low. Nevertheless, mycotoxin decontamination processes using microorganisms can be quite time-consuming [93].

**Figure 5:**
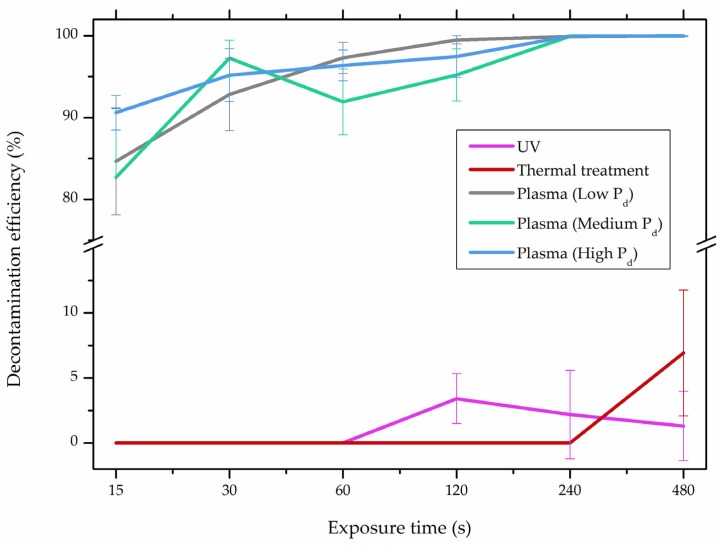
Comparison of decontamination efficiency (%) of aflatoxin B_1_ (AFB_1_) between cold atmospheric pressure plasma (CAP) and conventional decontamination approaches, UV light irradiation and thermal treatment; and air surface barrier discharge (SBD) plasma operated with three different discharge powers (P_d_; low P_d_, 10 W; med P_d_, 15 W; and high P_d_, 20 W). Ambient gas was used as a feed gas.

**Figure 6:**
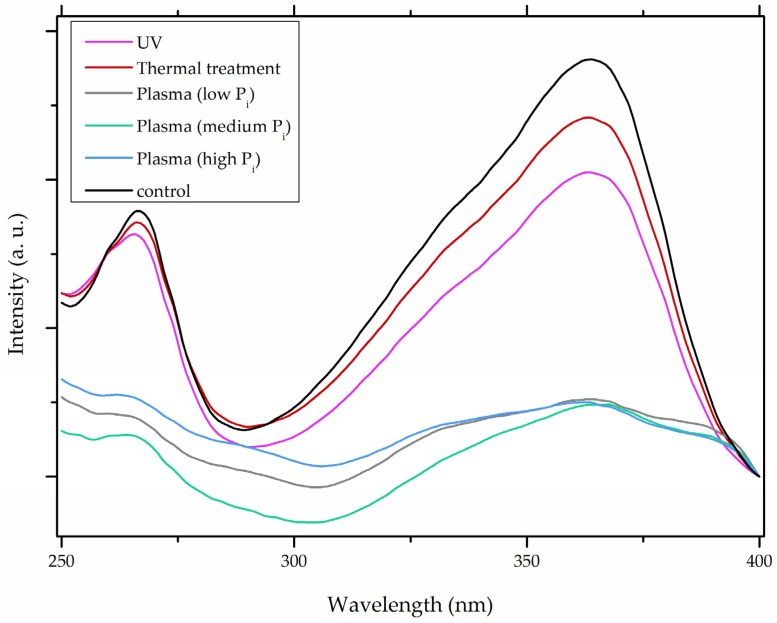
The comparison of aflatoxin B_1_ (AFB_1_) UV-Vis spectra after 8 min of exposure to heat treatment, UV irradiation and air surface barrier discharge (SBD) plasma operated with three different discharge powers (P_d_; low P_d_, 10 W; med P_d_, 15 W; and high P_d_, 20 W). Ambient gas was used as a feed gas.

In comparison with the methods described previously, CAP mycotoxin decontamination of food overcomes many of the disadvantages and obstacles of physical, chemical and microbial decontamination procedures. As depicted in Table 3, most of the CAP systems used for decontamination of food are environmentally benign, require a low energy input and are economically favourable. Beside this, plasma approaches have proven to have a negligible effect on the quality of many types of treated food. These advantages are based on the reactivity of the plasma species which enable the high decontamination efficiency in a very short time compared to alternative decontamination methods [46,53,105]. To demonstrate the efficiency of the plasma approach, a microwave-induced atmospheric pressure plasma system was used with argon as a carrier gas to treat three different mycotoxins, AFB_1_, DON, and NIV dried on glass coverslips. The treatment resulted in the complete decontamination of all three mycotoxins after only 5 s of plasma exposure. Plasma treatment completely eliminated their cytotoxicity as tested on mouse macrophage RAW264.7 cells in vitro [25]. Furthermore, low-temperature radiofrequency plasma was used to degrade AFB_1_. After 10 min of treatment, 88.3% AFB_1_ was degraded. Analysis of the degradation products indicated that the toxicity should be reduced based on the structure-activity criteria; the degradation pathways indicated the formation of five different decay products (Table 2), where plasma induced the loss of the double bond in the terminal furan ring (C_8_-C_9_) [26]. AFs were exposed to a dielectric barrier discharge (DBD) plasma system, resulting in the complete destruction of mycotoxins when they were treated alone. Using the same plasma system, a 70% decontamination level was achieved for the treatment of AFB_1_ contaminated dehulled hazelnuts [27]. To demonstrate the effectiveness of CAP, our recent experiments consider the use of an air surface barrier discharge (SBD) plasma treatment compared with UV light irradiation or thermal treatment in regards to AFB_1_-destruction efficiency. Standard solution of AFB_1_ was prepared in the mixture of acetonitrile and deionized water (2:1). One hundred microlitres of AFB_1_ standard solution was applied on the glass coverslips and dried for 5 min. Such wet samples were then exposed to CAP, UVC light, or thermal treatment. CAP set-up was similar to the one reported by Ni et al. [40] and was operated at three different discharge powers (P_d_): low (10 W), medium (15 W) and high (20 W). Low P_d_ operated plasma mostly contained ROS whereas RNS were the prevalent species at high P_d_ conditions. Plasma was observed to achieve more than 80% destruction level after just 15 s of treatment of AFB_1_ applied on, regardless of the P_d_ used. In contrast, no significant transformation of AFB_1_ was observed under thermal or UV light treatments, even at the longest exposure times (Figure 5). The ability of plasma to rapidly affect the AFB_1_ molecular structure was confirmed by UV-Vis spectrometry. As evident from Figure 6, both major peaks in the UV-Vis spectra of AFB_1_ significantly changed after 8 min of exposure of AFB_1_ to plasma, independently of the P_d_. On the other hand, the AFB_1_ UV-Vis spectra remained almost the same following the UV or thermal treatment for the same time period. UV light irradiation treatment is usually efficient in degrading only the mycotoxin molecules, in particular AFs, which are known for their photosensitivity [74,75,76]. Beside this, UV irradiation represents one of the most commonly used decontamination approaches in food processing [106]. Comparing to CAP, to achieve adequate results, this method requires much longer exposure times (more than 10 min compared to some seconds in the case of CAP). Here, it is worth mentioning that UV requires higher power inputs which further impacts the decontamination efficiency. In addition to the mentioned drawbacks, it has been reported that UV irradiation could even increase the mutagenicity of AFs [107]. The characteristics of mycotoxin plasma treatment can be compared to some extent with ozone treatment, since one of the prevalent plasma-produced long-lived molecular species is ozone [108]. As many other reactive species beside ozone are produced in the plasma, synergistic effects can occur, resulting in the mycotoxin decontamination of food requiring significantly less exposure times than ozone alone [48,84,85]. Despite numerous advantages, CAP technology also has some limitations. One of the major problems is an inability to precisely control the gas phase chemistry when using ambient air, given that it varies with conditions in the surrounding atmosphere (for example increases in humidity). Since CAP contains ROS, it is not suitable for the treatment of high-fat food products. Furthermore, when carried out using very high voltages, additional safety measures are required as well as systems for the destruction and exhaust of potentially harmful long-lived species such as O_3_ and NO_2_ [46].

## 4. Conclusions

Mycotoxin contaminated food represents a significant and increasing threat to human health and an enormous burden for the global economy. Decontamination methods to tackle this problem are based on physical, chemical and biological principles. In spite of constant improvements, these methods can still suffer from a lack of mycotoxin removal efficiency, they can be environmentally harmful and economically unfavourable. With no doubt, the food industry continuously strives for more effective mycotoxin decontamination approaches.

One of the most promising new procedures to deactivate mycotoxins on food is CAP technology. On the laboratory level, it has been convincingly demonstrated that CAP efficiently kills fungi on the surface of food and destroys the mycotoxins that these organisms secrete. In favour over many of the traditional food decontamination methods, plasma-based decontamination methods are generally lower-cost and ecologically benign. Most importantly, plasma-based mycotoxin decontamination of food has been demonstrated significantly more efficient in both the mycotoxin degradation level and speed of decontamination in comparison to conventional decontamination methods, as presented for the case of one of the most toxic mycotoxins, AFB_1_.

Before industrialization of CAP technology can be realised, the molecular mechanisms and kinetics of plasma-based mycotoxin decontamination should be better characterized in order to become standardized. For this reason, additional experimental work is needed to:

- Draw firm correlations between different plasma operating parameters and the specific reactive chemical species formed.
- Draw correlations between the composition of the plasma and the structure of the mycotoxin degradation products. As toxicities of the mycotoxin degradation products can be experimentally determined, in this way, the mycotoxin decontamination efficiency would be defined as well.
- Examine the effects of different plasma treatments on the quality of food products, for example on their nutritional value and organoleptic qualities.
- Design plasma-forming systems for efficient mycotoxin decontamination of various types and sizes of food products.
- Test if hybrid plasma-conventional systems for mycotoxin decontamination of food products can be even more effective.

## Figures and Tables

**Table 1:** Overview of the main characteristics of the most important mycotoxins.

Type	Representatives	Producing Fungi	Contaminated Foods	Structure Type	Toxicity
Aflatoxins (AF)	AFB_1_, AFB_2_, AFG_1_, AFG_2_, AFM_1_	*Aspergillus* spp.: *A. flavus* *A. parasiticus* *A. nomius* *A. bombycis* *A. pseudotamari* *A. ochraceoreus*	Crops, cereals, seeds, nuts, spices	Difuranocoumarins	Carcinogenicity
Ochratoxins (OT)	OTA, OTB, OTC	*Aspergillus* spp. and *Penicillium* spp.: *A. ochraceus* *A. aliaceus* *A. auricomus* *A. carbonarius* *A. glaucus* *A. meleus* *A. niger* *P. nordicum* *P. verrucosum*	Crops, fruits, beer, wine, juices, coffee	Polyketide-derived dihydroisocoumarins bound to L-β-phenylalanin by amid bond	Nephrotoxicity, mutagenicy, carinogenicity
Fumonisins	Series A (FA), B (FB), C (FC) and P (FP) with FB being the most common representatives: FB_1_, FB_2_, FB_3_	*Fusarium* spp.: *F. verticillioides* *F. proliferatum* *F. Napiforme* *F. dlamini* *F. nygamai*	Maize and its products	1, 2, 3-propanetricar-boxylic acid	Cytotoxicity, carcinogenicity
Zearalenone (ZEN)	ZEN, a-zearalenol, b-zearalenol	*Fusarium* spp.: *F. graminearum* *F. culmorum* *F. cerealis* *F. equiseti* *F. verticillioides* *F. incarnatum*	Crops, cereals	6-(10-Hydroxy-6-oxo-trans-1-undecenyl)-β-resorcylic acid lactone	Endocrine disruption
Trichothecenes	Deoxynivenol (DON), nivalenol (NIV), T-2 toxin, HT-2 toxin, diacetoxyscirpenol (DAS)	*Fusarium* spp. *Myrothecium* spp. *Phomopsis* spp. *Stachybotrys* spp. *Trychoderma* spp. *Trichotecium* spp. *Verticimonosporium* spp.	Crops	Tetracyclic-12,13-epoxy trichothenes	Inhibition of eucaryotic DNA, RNA and protein synthesis; nausea, vomiting, diarrhea, weight loss and loss of appetite, skin inflammation, vomiting, liver damage
Ergot alkaloids (EAs)	Ergometrine, ergotamine, ergosine, ergocristine, ergocryptine, ergocornine and the corresponding –inine epimers	*Claviceps* spp.: *C. purpurea*	Grains, grass	Tetracyclic ergolines (tryptophan-derived alkaloids)	Neurotoxicity, endocrine disruption
Other mycotoxins	Fusaproliferin (FUS), enniatins (ENNs), beauvericin (BEA), moniliformin (MON), patulin (PAT)	*Fusarium* spp. *Penicillium* spp. *Aspergillus* spp. *Eupenicillium* spp. *Paecilomyces* spp. *Byssochlamys* spp.	Crops, fruits, vegetables, cereals	Sesterterpene cyclic hexadepsipeptides, 3-hydroxycyclobut-3-ene-1,2-dione, 4-hydroxy-4H-furo[3,2-c]pyran-2(6H)-one	Cytotoxicity, abnormal gluconeiogenesis, genotoxicity and mutagenicity

**Table 2:** Degradation products of AFB_1_ after treatment with different decontamination methods.

Decontamination Method	Degradation Products	Reference
UV	In aqueous solution: 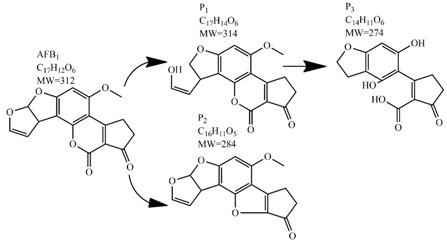	[75]
	In acetonitrile: 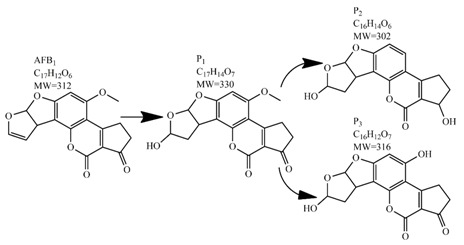	[76]
	In peanut oil: 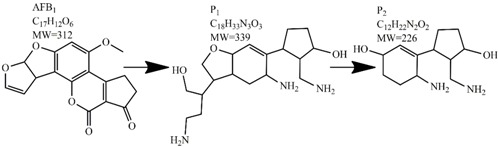	[74]
Plasma	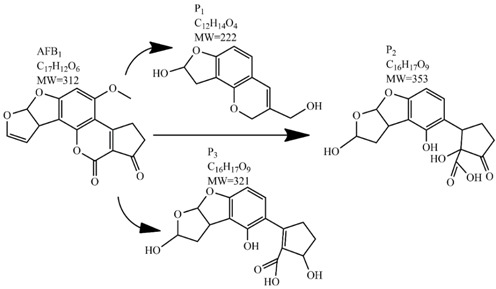	[26]
	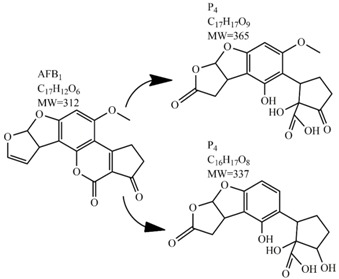	
Ozone	In acetonitrile: 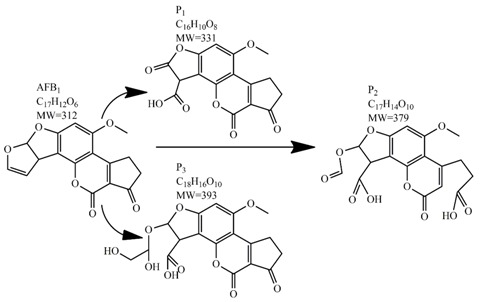	[85]
	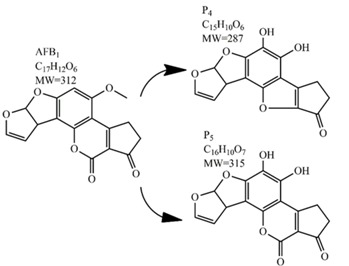	
Ammoniation	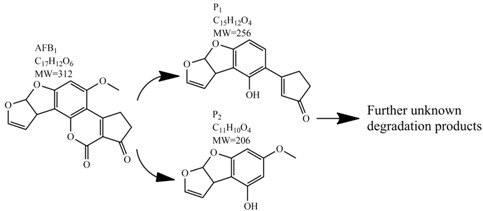	[81]
*P. putida*	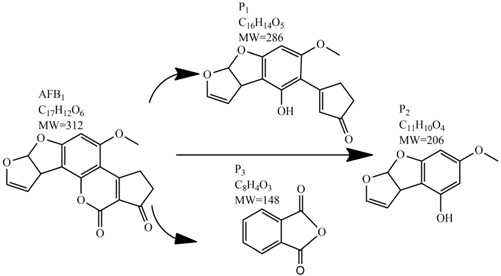	[96]

**Table 3:** The comparison between mycotoxin decontamination methods.

Decontamination Method	Highest Decontamination Rate Obtained	Food Product	Process Duration	Energy Consumption	Impact on the Food Quality	Reference
Thermal treatment	85–100% (FBs, ZEN, AFs)	Corn	Long	High	Significant	[70,109,110]
Gamma irradiation	90% (mixture)	Grains, seeds	Short	Low	Significant	[71]
UV light irradiation	90% (AFB_1_, PAT)	Peanut oil; apple juice;	Short	Low	Negligible	[74,77]
Pulsed light technology	90% (AFB_1_)	Rice products	Short	Low	Negligible	[79]
Ammoniation	90–100% (AFB_1_)	Rice	Long	High	Significant	[83]
Ozonation	80% (AFs)	Corn flour, peanuts	Long	Low	Negligible	[84,85]
*Bacillus* spp.	92.5% (OTA)	/	Long	Low	Negligible-significant	[111]
*Rhodococcus erythropolis*	90% (AFB_1_)	/	Long	Low	Negligible	[98]
*Aspergillus* spp.	100% (ZEN)	/	Long	Low	Negligible-significant	[99]
*Trichosporon mycotoxinivorans*	100% (OTA)	Animal Feed	Long	Low	Negligible	[103]
Lactic acid bacteria	80–100% (FBs)	/	Long	Low	Negligible	[112]
CAP technology	100% (AFs, DON, NIV)	Seeds, crops, cereals	Short	Low	Negligible	[25,27]
